# *Sclerosperma* fossils from the late Oligocene of Chilga, north-western Ethiopia

**DOI:** 10.1080/00173134.2018.1510977

**Published:** 2018-10-25

**Authors:** Friðgeir Grímsson, Bonnie F. Jacobs, Johan L. C. H. Van Valkenburg, Jan J. Wieringa, Alexandros Xafis, Neil Tabor, Aaron D. Pan, Reinhard Zetter

**Affiliations:** 1 Department of Palaeontology, University of Vienna, Vienna, Austria; 2 Roy M. Huffington Department of Earth Sciences, Southern Methodist University, Dallas, TX, USA; 3 Naturalis Biodiversity Center, National Herbarium of The Netherlands, Leiden, The Netherlands; 4 Don Harrington Discovery Center, Streit Drive, TX, USA

**Keywords:** Arecaceae, palm leaf, light microscopy, palaeovegetation, palms, pollen morphology, rainforest, scanning electron microscopy, swamp element

## Abstract

The palm family, Arecaceae, is notoriously depauperate in Africa today, and its evolutionary, paleobiogeographic, and extinction history there are not well documented by fossils. In this article we report the pollen of two new extinct species of the small genus, *Sclerosperma* (Arecoideae), from a late Oligocene (27–28 Ma) stratum exposed along the Guang River in Chilga Wereda of north-western Ethiopia. The pollen are triporate, and the two taxa can be distinguished from each other and from modern species using a combination of light and scanning electron microscopy, which reveals variations in the finer details of their reticulate to perforate exine sculpture. We also report a palm leaf fragment from a stratum higher in the same section that is in the Arecoideae subfamily, and most likely belongs to *Sclerosperma*. The implications of these discoveries for the evolutionary history of this clade of African arecoid palms is that their diversification was well underway by the middle to late Oligocene, and they were much more widespread in Africa at that time than they are now, limited to West and Central Africa. *Sclerosperma* exhibits ecological conservatism, as today it occurs primarily in swamps and flooded forests, and the sedimentology of the Guang River deposits at Chilga indicate a heterogeneous landscape with a high water table. The matrix containing the fossil pollen is lignite, which itself indicates standing water, and a variety of plant macrofossils from higher in the section have been interpreted as representing moist tropical forest or seasonally inundated forest communities.

*Sclerosperma* G. Mann et H. Wendl. is a small genus in the palm family, Arecaceae (Arecoideae, Sclerospermeae; Dransfield et al. 2005), found in tropical West and Central Africa where three species are known; *Sclerosperma mannii* H. Wendl., *S. profizianum* Valk. et Sunderl. and *S. walkeri* A. Chev. (van Valkenburg et al. 2008). The genus was first described by Gustav Mann and Hermann Wendland in 1864 based on material belonging to *S. mannii* collected by Mann in an inundated forest near the Gaboon (now the Ogooué) River upstream from Point Clara (Mann & Wendland 1864). But, *Sclerosperma* remained rather enigmatic over time, and although new species were described, they were so rarely collected that the circumscription of these taxa was clarified only recently (van Valkenburg et al. 2008). All three species of *Sclerosperma* are small, clustering understorey palms, ranging in height from 2 to 6 (to 12) m. They are vulnerable, pleonanthic, monoecious palms that grow in tropical lowland rainforests (from sea level to *c*. 1400 m), on swampy sites as well as, less commonly, on terra firma. *Sclerosperma* is present in old secondary forests dominated by okoumé (*Aucoumea klaineana* Pierre) and persists in secondary growth near human habitations where the leaves are often collected for roof thatch. The stem in *Sclerosperma* is usually absent above-ground, is stout with closely-spaced ringed leaf scars, and if above-ground may extend horizontally and produce suckers. Occasionally, *S. profiziana* can produce a stout stem up to 9 cm in diameter and 2 m in height. The crown consists of numerous leaves, broadly radiating, pointing upwards, that accumulate debris, and thus obscure the base of the plant. The leaves are reduplicate, undivided or irregularly pinnate, the apex deeply bifid with the rachis continued in a fibre. All *Sclerosperma* species have intrafoliar, solitary, spike-like inflorescences, which are protogynous. The inflorescence is enclosed by a peduncular bract which becomes web-like in the median part at anthesis and with a distal opening. This peduncular bract is somewhat persistent but usually disintegrates when fruits are fully developed. The inflorescence is often obscured by debris, can be overlooked, and thus rarely collected. The fruits are readily sought by animals (e.g. gorillas), so in faunal-rich regions intact infructescences are rarely found (van Valkenburg et al. 2008). The unique pollen morphology of *Sclerosperma* (triangular, triporate, reticulate) within the Arecaceae was first noted by Erdtman and Sing (1957) and often discussed by M.M. Harley and collaborators in the years 1991 to 2008 during their comprehensive work on the pollen morphology of this family (Harley & Hall 1991; Harley 1996, 1999, 2004; Harley & Baker 2001; Harley & Dransfield 2003; Dransfield et al. 2008). The pollen morphology of all three extant *Sclerosperma* species was recently revised by Grímsson et al. (2018), showing that they produce four different pollen morphologies. *S. mannii* and *S. walkeri* share the same distinct reticulate pollen, but *S.profizianum* appears to produce three different pollen types (microreticulate, fossulate, and perforate).

The fossil record of *Sclerosperma* is meagre, and is only represented by leaf specimens from a site called ‘Mero’, near Kamituga, in eastern Democratic Republic of the Congo (DRC), South Kivu District. Lakhanpal (1966) described and figured *S*. *saffiannikoffii* from this locality, which was referred to as middle Miocene in age. The fossil pollen record, so far, has mostly been questioned or rejected by Harley and Baker (2001) and Harley (2004, 2006). Nevertheless, the fossil record of Arecaceae in Africa can help to understand its depauperate representation today relative to other tropical regions of the world (e.g. 65 species in Africa versus 437 in South America), and to assess the relative importance of Cainozoic extinctions versus moderate speciation rates compared with other, richer, regions (Richards 1973; Morley 2000; Pan et al. 2006; Couvreur et al. 2011; Kissling et al. 2012; Couvreur & Baker 2013; Faye et al. 2016).

Here we describe fossil *Sclerosperma* pollen, assigned to two new species, from the late Oligocene of Chilga, north-western Ethiopia. The fossil pollen is compared to that of extant *Sclerosperma* taxa in light of recent descriptions presented by Grímsson et al. (2018), and the evolution of *Sclerosperma* pollen morphology is discussed. The fossil pollen record is summarised, and conclusions are drawn regarding the palaeophytogeographic history of the genus, African palms in general, and the significance of *Sclerosperma* for palaeoenvironmental interpretations of the Chilga site. We also report an unidentified palm leaf fragment from the same site that can be assigned to Arecoideae, and although it was found at a different stratigraphic level could belong to a *Sclerosperma* taxon described herein.

## Material and methods

### Geologic background of sedimentary sample

**Figure 1.**
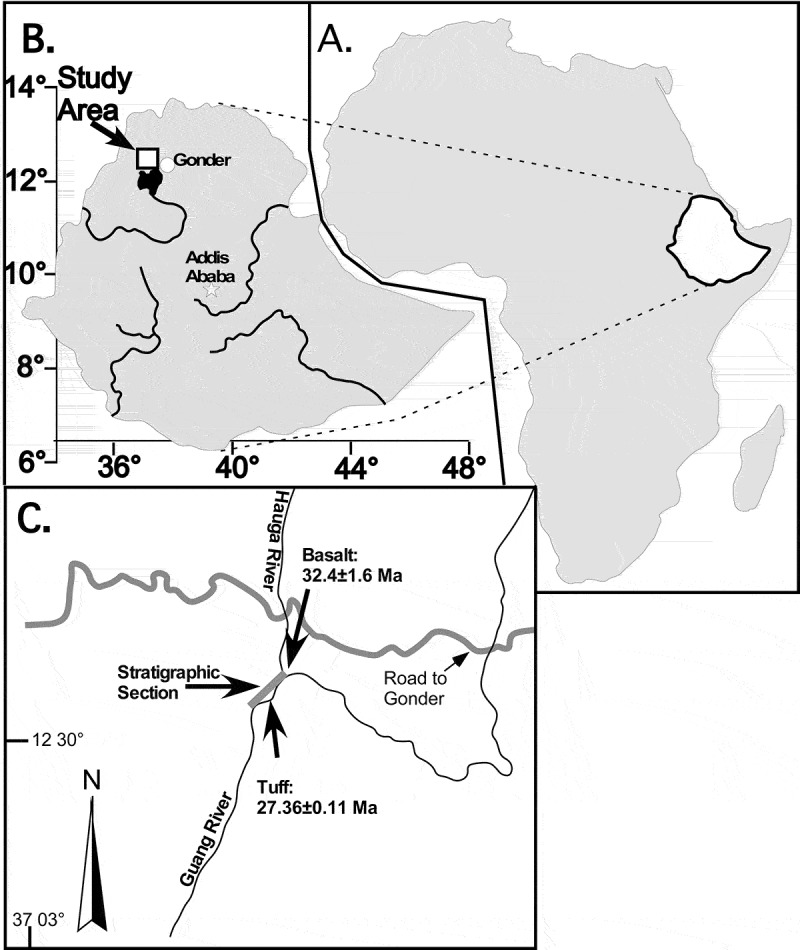
Locality map. **A.** Location of Ethiopia in Africa. **B.** The study area, Gonder, and the current capital city, Addis Ababa. Major rivers are shown, including the Blue Nile and its source, Lake Tana, to the immediate south of the study area. **C.** Location of the Guang and Hauga Rivers, and measured section shown in Figure 2.

**Figure 2.**
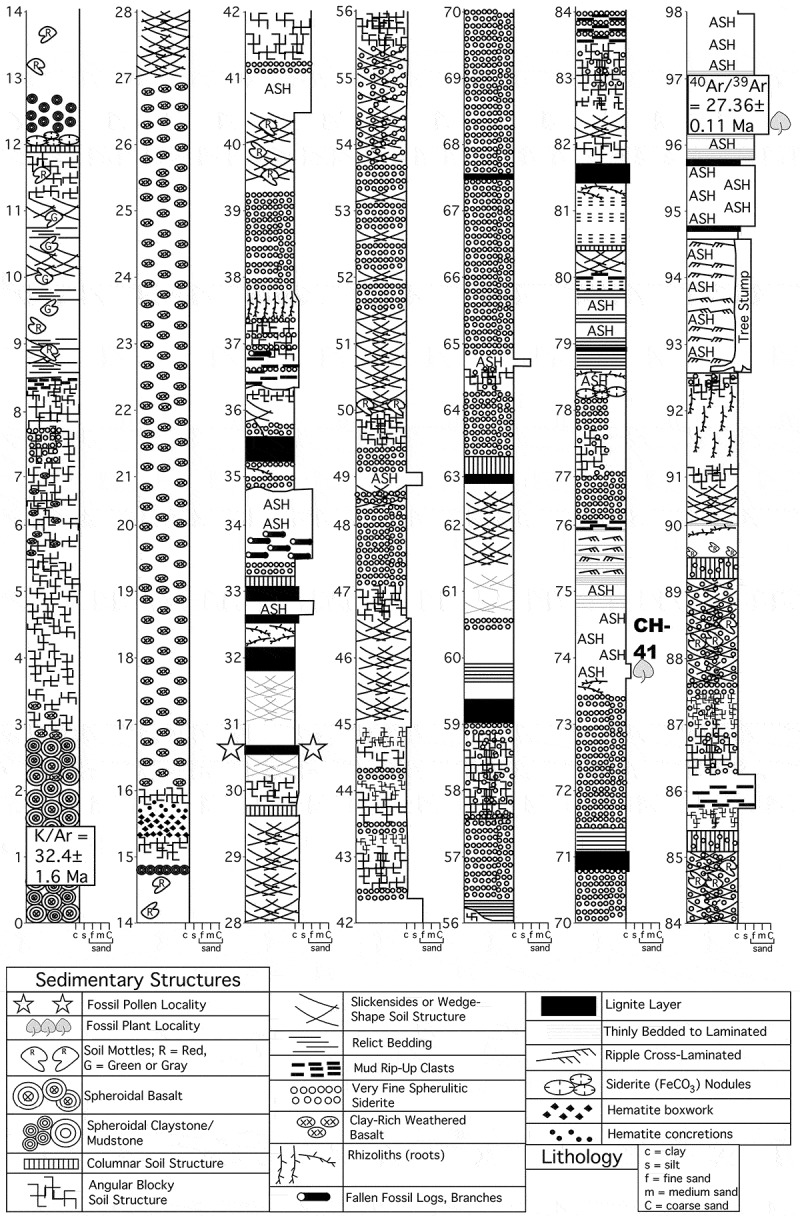
Stratigraphic section, measured along the Guang River. Stars indicate stratum that produced the *Sclerosperma* fossil pollen, and the fossil leaf locality, CH41, is labelled and marked by a leaf icon. Radioisotopic dates are shown near the base and top of the section.

The *Sclerosperma* fossils, both pollen and leaves, were found in sedimentary rocks from the late Oligocene of Chilga deposits of north-western Ethiopia (Figure 1). Fossil rich sedimentary rocks outcrop in an area of about 100 km^2^, approximately 60 km to the west-southwest (WSW) of Gonder, the historic capital of Ethiopia. Physiographically, this region constitutes a part of the Ethiopian Highlands to the west of the main Ethiopian Rift, and lies at elevations of 1800 to 2000 m. The Chilga sedimentary sequence, >100 m thick, occurs in a structural basin underlain by basalts of early Oligocene age. A volcanic ash layer near the top of the Chilga section provided a ^40^Ar–^39^Ar age of 27.36 ± 0.11 Ma, and the underlying basalts associated with the origin of the Ethiopian Plateau is dated by whole-rock K-Ar at 32.4 ± 0.11 Ma (Figure 2; Kappelman et al. 2003). The sedimentary rocks are also correlated with the paleomagnetic time scale, constraining the age of the fossiliferous strata to 28–27 Ma, within the limits of Chron C9n (Kappelman et al. 2003). Fossil vertebrates and plants were collected widely across the Chilga outcrop area, but plant macrofossils and microfossils were studied most intensively from localities exposed along the Guang River and its smaller tributary, the Hauga River (Figure 1; e.g. Yemane et al. 1985, 1987; Kappelman et al. 2003; Sanders et al. 2004; Jacobs et al. 2005; Pan & Jacobs 2009; Engel et al. 2013; Pan et al. 2014). The sedimentary sample that produced the pollen reported here, is a lignite from 30.5 m above the base of the measured section along the Guang River (Figure 2), and the fossil palm leaf fragments were found in an ash-rich siderite approximately 43 m higher in the section (74 m, Figure 2).

### Preparation of fossil pollen

The sedimentary rock sample was treated with hydrochloric acid (HCl) and hydrofluoric acid (HF) to remove any carbonates and silicates, followed by an oxidising solution, according to the protocol outlined in Grímsson et al. (2008). The fossil *Sclerosperma* pollen grains were investigated both by light microscopy (LM) and scanning electron microscopy (SEM) using an isolated single grain as described in Zetter (1989) and Halbritter et al. (2018). Plant macrofossils were collected in blocks of matrix from four closely-spaced sublocalities along a single, organic rich stratum, mostly lacking bedding planes or laminations. The mode of deposition is interpreted as overbank associated with a flood event.

### Conservation of fossil material

SEM stubs with the fossil *Sclerosperma* pollen produced during this study are stored in the collection of the Department of Palaeontology, University of Vienna, Austria, under the accession numbers IPUW 7513/223 to IPUW 7513/229. The fossil leaves are housed permanently in the Chilga paleobotany collections of the National Museum of Ethiopia, Addis Ababa, under accession numbers CH41-9A and CH41-9B (A and B are part and counterpart).

## Systematic and descriptive palaeobotany

Pollen descriptions include diagnostic features observed both in LM and SEM. The pollen terminology follows Punt et al. (2007; LM) and Halbritter et al. (2018; SEM). Classification follows Dransfield et al. (2005) and APG IV  (2016). The pollen grains are described first, followed by the leaf remains. The fossil pollen types are compared to pollen of extant taxa in Table I. Terminology for palm leaf morphology follows Hickey (1973), Ellis et al. (2009), and especially Dransfield et al. (2008).

**Table I.** Pollen morphology of extant and fossil *Sclerosperma.*

	*S. mannii*	*S. protomannii* sp. nov.	*S. walkeri*	*S. profizianum* (Type A)	*S. protoprofizianum* sp. nov.	*S. profizianum* (Type C)	*S. profizianum* (Type B)
Distribution	Liberia, Nigeria, Cameroon, Equatorial Guinea, Gabon, Angola, Democratic Republic of the Congo (DRC)	Chilga, north-western Ethiopia	Gabon, DRC	Ghana, Gabon, Republic of the Congo, DRC, Angola (species distibution)	Chilga, north-western Ethiopia	Ghana, Gabon, Republic of the Congo, DRC, Angola (species distibution)	Ghana, Gabon, Republic of the Congo, DRC, Angola (species distibution)
Age	Recent	Late Oligocene	Recent	Recent	Late Oligocene	Recent	Recent
Outline polar view	Straight-triangular to slightly concave-triangular	Straight-triangular to slightly concave-triangular	Straight-triangular to slightly concave-triangular	Straight-triangular to slightly concave-triangular	**Straight-triangular; apices often infolded towards proximal side (hexagonal appearance)**	Straight-triangular to slightly convex-triangular	Straight-triangular to slightly concave-triangular
Outline equatorial view	Bean-shaped	Bean-shaped	Bean-shaped	Bean-shaped	Bean-shaped	Bean-shaped	Bean-shaped
Equatorial diameter (µm in LM)	32–38	27–38	35–40	35–40	**23–35**	37–42	32–38
Equatorial diameter (µm in SEM)	27–34	24–35	30–35	29–35	**21–29**	31–39	30–34
Polar axis (µm in LM)	9–15	9–12	15–19	10–14	9–12	10–16	10–13
Aperture type	Triporate	Triporate	Triporate	Triporate	Triporate	Triporate	Triporate
Aperture position	Sub-apically, distal polar face	Sub-apically, distal polar face	Sub-apically, distal polar face	Sub-apically, distal polar face	Sub-apically, distal polar face	Sub-apically, distal polar face	Sub-apically, distal polar face
Aperture outline	Elliptic	Elliptic	Elliptic	Elliptic	Elliptic	Circular to elliptic	Circular to elliptic
Aperture diameter (µm in SEM)	4.5–6.0	5.5–8.0	5.0–8.0	5.0–8.5	6.0–8.0	4.0–6.5	4.0–5.5
Exine thickness (µm in LM)	1.7–2.5	1.7–2.5	1.7–2.5	1.7–2.5	1.7–2.5	1.7–2.5	1.7–2.5
Pollen wall (SEM)	Semitectate	Semitectate	Semitectate	Semitectate	**Semitectate to tectate**	Tectate	Tectate
Sculpture (LM)	Reticulate	Reticulate	Reticulate	Reticulate	Reticulate	Scabrate	Rugulate
Sculpture (SEM)	Reticulate to perforate	Reticulate to perforate	Reticulate to perforate	Microreticulate to perforate	Microreticulate to perforate	Perforate, rugulate/verrucate and fossulate	Fossulate, rugulate/verrucate, perforate
Sculpture distal face (SEM)	Reticulate with broad muri and elliptic to triangular to polygonal lumina, 0–6 nanogemmae free-standing columellae per lumina	Reticulate with broad muri and elliptic to polygonal lumina	Reticulate with broad muri and elliptic to triangular to polygonal lumina, 0–6 nanogemmae free-standing columellae per lumina	Microreticulate with broad muri and elliptic to triangular to polygonal lumina	**Microreticulate to perforate** with broad muri and elliptic to polygonal lumina or circular to slit-like perforations, **free-standing columellae rare, up to 4 nanogemmae free-standing columellae per lumina when present**	Perforate, perforations elliptic to slit-like, perforations often aligned in sinuous rows, rows of perforations outlining irregular shaped rugulae/verrucae	Fossulate with tiny circular to slit-like perforations aligned within the fossulae, sinuous fossulae outlining irregular shaped rugulae/verrucae
Number of lumina/perforations at central distal face (SEM)	18–25 per 100 µm^2^	**10–20 per 100 µm^2^**	16–25 per 100 µm^2^	30–35 per 100 µm^2^	**50–65 per 100 µm^2^**	45–55 per 100 µm^2^	Not applicable
Sculpture proximal face (SEM)	Reticulate central polar area and mesoporium with elliptic to triangular to polygonal lumina, 0–6 nanogemmae free-standing columellae per lumina; becoming microreticulate to perforate towards apices	Reticulate central polar area and mesoporium with **elliptic to polygonal lumina, nanogemmae free-standing columellae extremely rare and singular**; becoming microreticulate to perforate towards apices	Reticulate central polar area and mesoporium with elliptic to triangular to polygonal lumina, 0–6 nanogemmae free-standing columellae per lumina; becoming microreticulate to perforate towards apices	Microreticulate central polar area and mesoporium with elliptic to circular or slit-like lumina; becoming nanoreticulate to perforate towards apices	**Microreticulate to perforate** central polar area and mesoporium **with elliptic to circular to polygonal or slit-like lumina/perforations, free-standing columellae rare, up to 4 nanogemmae free-standing columellae per lumina when present**; becoming nanoreticulate to perforate towards apices	Perforate and fossulate central polar area and mesoporium, perforations elliptic to slit-like, perforations often aligned in sinuous rows, rows of perforations and fossulae outlining irregular shaped rugulae/verrucae; becoming micro- to nanorugulate/verrucate and perforate towards apices	Fossulate central polar area and mesoporium with tiny circular to slit-like perforations aligned within the fossulae, sinuous fossulae outlining irregular shaped rugulae/verrucae; becoming micro- to nanorugulate/verrucate and perforate towards apices
Opercula (SEM)	Nanoverrucate to granulate sublayer and microreticulate supra-layer	Sublayer not observed, but with **reticulate** supra-layer	Nanoverrucate to granulate sublayer and microreticulate supra-layer	Nanoverrucate to granulate sublayer and reticulate supra-layer	Nanoverrucate to granulate sublayer and **microreticulate** supra-layer	Nanoverrucate to granulate sublayer and perforate supra-layer	Nanoverrucate to granulate sublayer and perforate supra-layer

Note: Data on extant pollen from Grímsson et al. (2018). The fossil *Sclerosperma* pollen types are placed between those extant taxa they mostly compare to.

All measurements are given in micrometres (µm). Most diagnostic features separating the fossil pollen appear in bold font.

Family Arecaceae Bercht. et J. Presl

*Genus* Sclerosperma *G. Mann et H. Wendl.*

**Figure 3.**
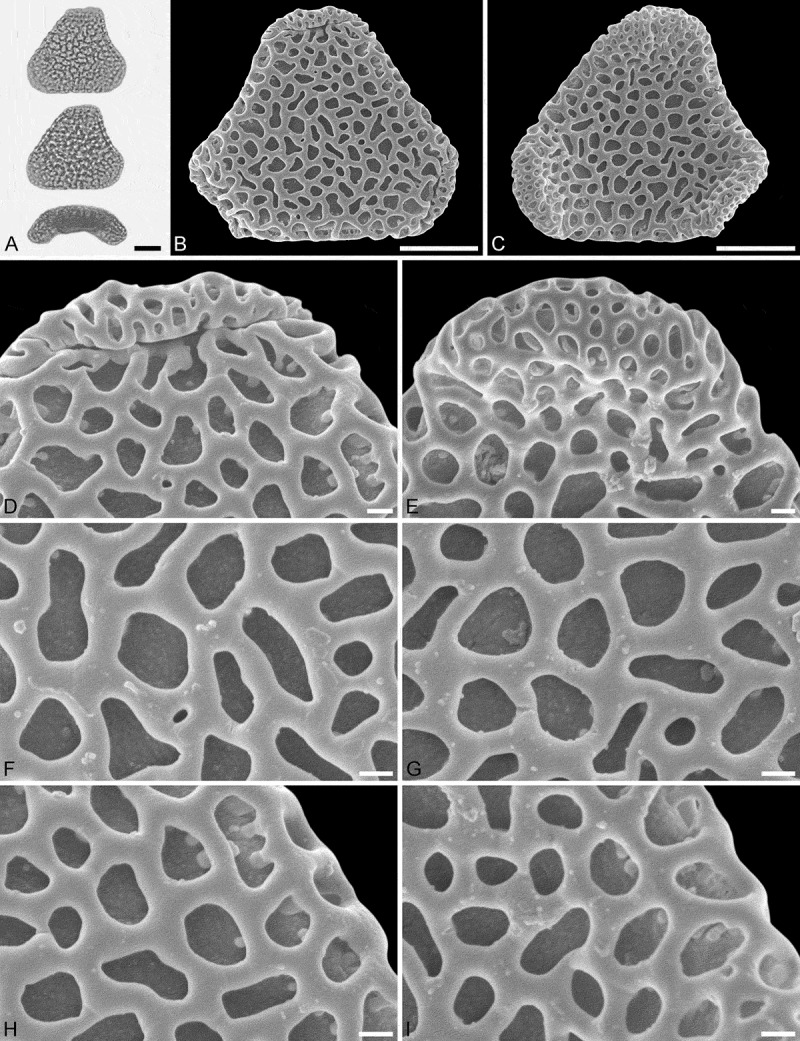
Light microscopy (LM) (**A**) and scanning electron microscopy (SEM) (**B**–**I**) micrographs of *Sclerosperma protomannii* sp. nov. (holotype: IPUW 7513/223). **A.** Pollen grain in polar view (upper in high focus and middle in optical cross-section) and equatorial view (lower). **B.** Pollen grain in polar view, distal side. **C.** Pollen grain in polar view, proximal side. **D.** Close-up of apex with aperture, distal side. **E.** Close-up of apex, proximal side. **F.** Close-up of central polar area, distal side. **G.** Close-up of central polar area, proximal side. **H.** Close-up of interapertural area, distal side. **G.** Close-up of interapertural area, proximal side. Scale bars – 10 µm (A–C), 1 µm (D–I).

**Figure 4.**
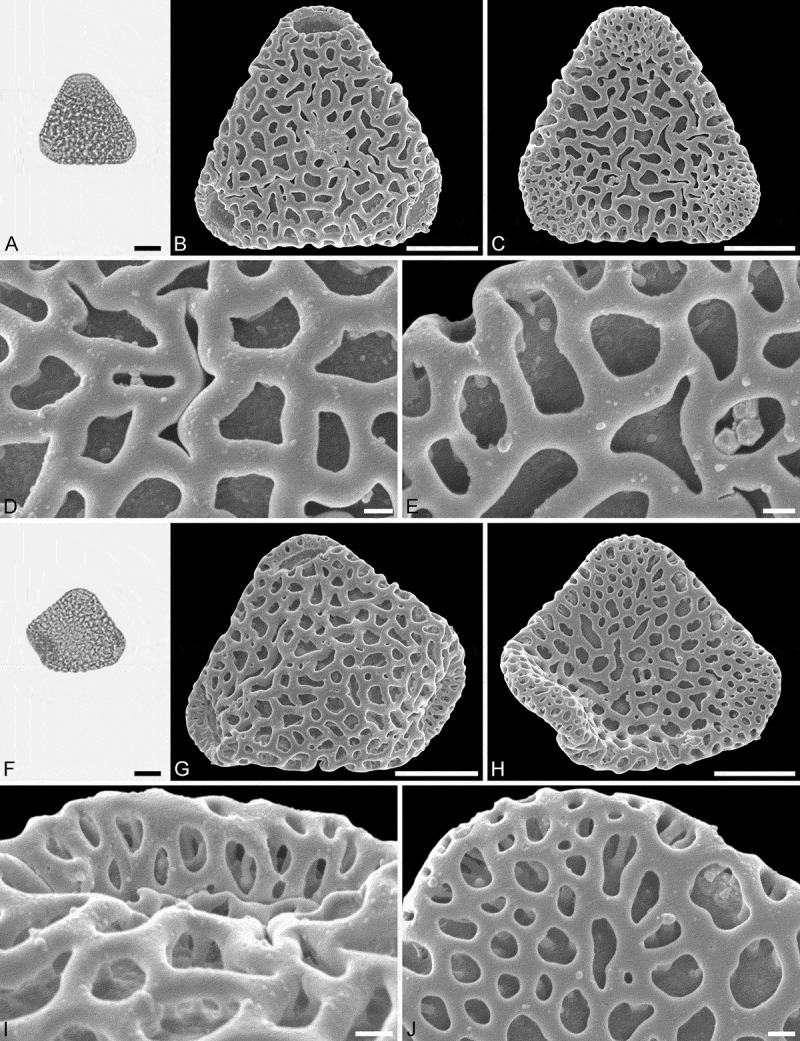
Light microscopy (LM) (**A**) and scanning electron microscopy (SEM) (**B**–**E**) micrographs of *Sclerosperma protomannii* sp. nov. (paratype: IPUW 7513/224). **A.** Pollen grain in polar view (optical cross-section). **B.** Pollen grain in polar view, distal side. **C.** Pollen grain in polar view, proximal side. **D.** Close-up of central polar area, distal side. **E.** Close-up of interapertural area, proximal side. LM (**F**) and SEM (**G**–**J**) micrographs of *S*. *protomannii* sp. nov. (paratype: IPUW 7513/225). **F.** Pollen grain in polar view (high focus). **G.** Pollen grain in polar view, distal side. **H.** Pollen grain in polar view, proximal side. **I.** Close-up of apex with aperture, distal side. **J.** Close-up of apex, proximal side. Scale bars – 10 µm (A–C, F–H), 1 µm (D, E, I, J).

**Figure 5.**
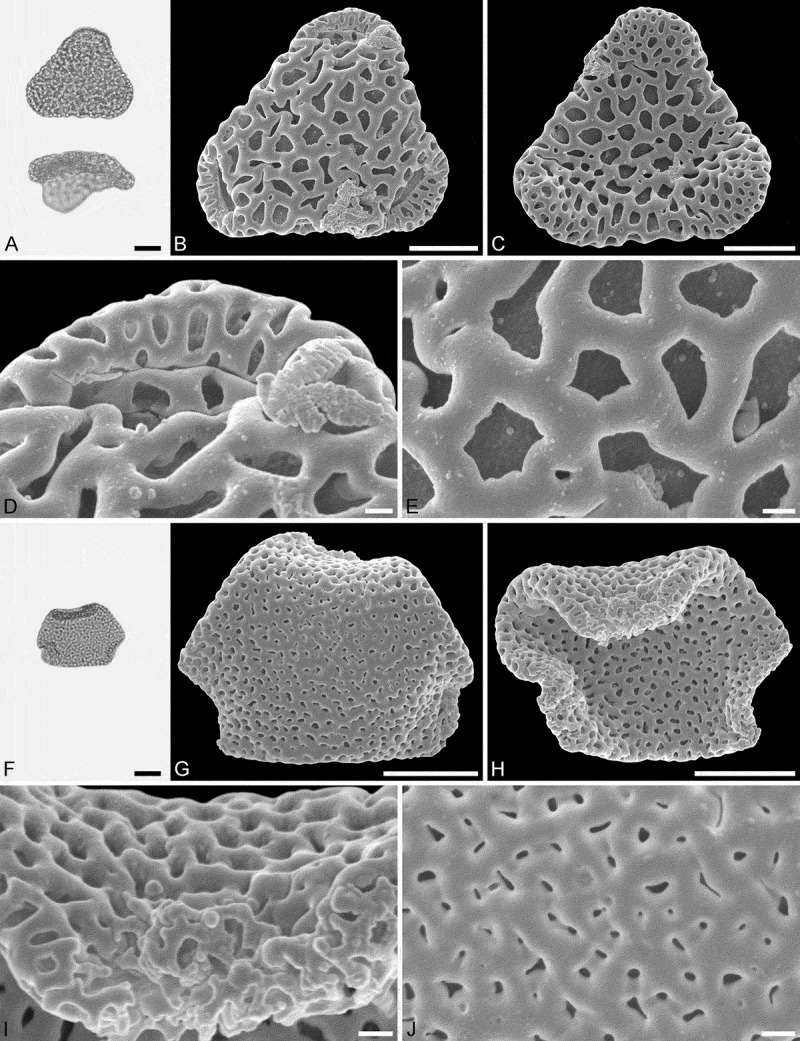
Light microscopy (LM) (**A**) and scanning electron microscopy (SEM) (**B**–**E**) micrographs of *Sclerosperma protomannii* sp. nov. (paratype: IPUW 7513/226). **A.** Pollen grain in polar view (upper, high focus) and equatorial view (lower). **B.** Pollen grain in polar view, distal side. **C.** Pollen grain in polar view, proximal side. **D.** Close-up of apex with aperture, distal side. **E.** Close-up of central polar area, distal side. LM (**F**) and SEM (**G**–**J**) micrographs of *S*. *protoprofizianum* sp. nov. (holotype: IPUW 7513/227). **F.** Pollen grain in polar view (high focus). **G.** Pollen grain in polar view, distal side. **H.** Pollen grain in polar view, proximal side. **I.** Close-up of apex with aperture, distal side. **J.** Close-up of central polar area, distal side. Scale bars – 10 µm (A–C, F–H), 1 µm (D, E, I, J).

*Species* Sclerosperma protomannii *Grímsson et R. Zetter, sp. nov.*

(Figures 3, 4, 5A–E)

### 

#### Diagnosis

Pollen with 10 to 20 lumina per 100 µm^2^ at central distal face, elliptic to polygonal lumina; nanogemmae free-standing columellae extremely rare and singular; opercula with a reticulate supra-layer. All other pollen features that can be observed under LM and SEM comparable to that observed in two of the modern species: *Sclerosperma mannii* and *S. walkeri*.

#### Holotype

IPUW 7513/223 (Figure 3).

#### Paratypes

PUW 7513/224 to IPUW 7513/226 (Figures 4, 5A–E).

#### Type locality

Guang River, Chilga, North Gondar Zone, Amhara Region, north-western Ethiopia; *c*. 12° 30′ N, 37° 7′ E.

#### Stratigraphy

Chilga strata (Figure 2).

#### Age

Late Oligocene, 28–27 Ma (Kappelman et al. 2003).

#### Species epithet

After the extant species, *Sclerosperma mannii*, that also shows a clear reticulate sculpture in SEM.

#### Description

Pollen, monad, heteropolar, polar/equatorial (P/E) ratio oblate, outline straight-triangular to slightly concave-triangular in polar view, bean-shaped in equatorial view (convex distal face versus concave proximal face); equatorial diameter 27–38 µm in LM, 24–35 µm in SEM, polar axis 9–12.5 µm in LM; triporate, pori positioned sub-apically on the distal polar face, pori elliptic, 5.5–8.0 µm in diameter, pori equipped with opercula; exine 1.7–2.5 µm thick in LM, nexine thinner than sexine; pollen wall semitectate; sculpture reticulate in LM, reticulate to perforate in SEM; distal face reticulate with broad muri and elliptic to polygonal lumina, 10–20 lumina per 100 µm^2^ at central distal face, nanogemmae free-standing columellae extremely rare and singular when present (SEM); proximal face reticulate to perforate, lumina/perforations elliptic to polygonal, nanogemmae free-standing columellae extremely rare and singular when present, central polar area and mesoporium reticulate, sculpture becoming microreticulate to perforate towards apices; opercula with a distinct reticulate supra-layer (SEM).

#### Remarks

The outline of this fossil pollen type in both polar and equatorial view, along with its reticulate sculpture, distally placed apertures and reticulate opercula, places it firmly within the extant genus *Sclerosperma* (Table I). Of the four pollen morphologies produced by extant *Sclerosperma* (see Grímsson et al. 2018), the *S. protomannii* sp. nov. pollen is most similar to that of *S. mannii* and *S. walkeri*. Both these taxa produce distinctly reticulate pollen with broad muri and elliptic to polygonal lumina. Still, the *S. protomannii* sp. nov. pollen is much smaller than the *S. walkeri* pollen and equal in size only to the smallest of the *S. mannii* pollen (Table I). The *S. protomannii* pollen has up to 20 lumina per 100 µm^2^ at the central distal pole, but both *S. mannii* and *S. walkeri* can have up to 25 lumina per 100 µm^2^. Also, *S. mannii* and *S. walkeri* pollen has up to six nanogemmae free-standing columellae per lumina, but the lumina in *S. protomannii* sp. nov. rarely have any free-standing columellae, and when present they are tiny and singular. Additionally, the operculum of *S. protomannii* sp. nov. has a reticulate supra-layer versus microreticulate in both *S. mannii* and *S. walkeri*.

Since this is the first fossil *Sclerosperma* pollen ever described using SEM, and the fossil pollen record for this genus is nearly non-existent (see Discussion; Table II), comparisons are limited. The only illustrated fossil *Sclerosperma* pollen grains, published by Medus (Medus 1975, plate 4, figures 2 and 3), are badly preserved, and in the one focus level provided it is hard to distinguish if the two LM micrographs show reticulate or rugulate sculpture.

**Table II.** Fossil record of *Sclerosperma*

Taxon	Organ	Locality	Age	Country	Reference
*Scleropserma* sp.	Pollen	Bignona borehole	Late Miocene (10–5 Ma)	Senegal	Medus (1975, plate 4, figures 2–3)
*Sclerosperma safiannikoffii*	Leaves, cuticle	Mero camp	?Miocene	Democratic Republic of the Congo	Lakhanpal (1966, plate 1, figure 1; plate 2, figure 1; plate 3, figures 1–2)

Note: This list includes all*Sclerosperma* records verified using illustrated material (pollen, leaves, and cuticle).

All other records have been excluded.

*Species* Sclerosperma protoprofizianum*Grímsson et R. Zetter, sp. nov.*

(Figures 5F–J, 6)

#### Diagnosis

Pollen straight-triangular in outline; semitectate to tectate pollen wall; microreticulate to perforate sculpture at distal face; 50–65 lumina per 100 µm^2^ at central distal face; elliptic to circular to polygonal or slit-like lumina/perforations; opercula with microreticulate supra-layer. Most other pollen features that can be observed under LM and SEM comparable to that observed in two pollen types (A and C) of the modern species: *Sclerospermaprofizianum*.

#### Holotype

IPUW 7513/227 (Figure 5F–J).

#### Paratypes

**Figure 6.**
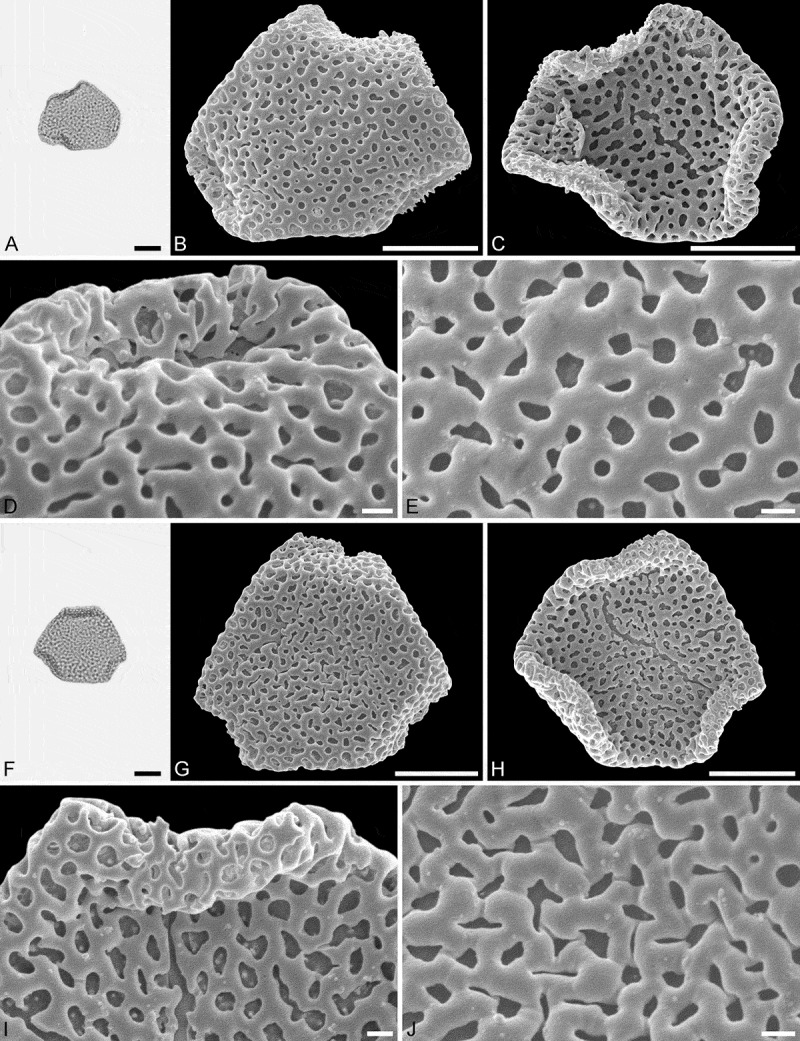
Light microscopy (LM) (**A**) and scanning electron microscopy (SEM) (**B**–**E**) micrographs of *Sclerosperma protoprofizianum* sp. nov. (paratype: IPUW 7513/228). **A.** Pollen grain in polar view (high focus). **B.** Pollen grain in polar view, distal side. **C.** Pollen grain in polar view, proximal side. **D.** Close-up of apex with aperture, distal side. **E.** Close-up of central polar area, distal side. LM (**F**) and SEM (**G**–**J**) micrographs of *S*. *protoprofizianum* sp. nov. (paratype: IPUW 7513/229). **F.** Pollen grain in polar view (high focus). **G.** Pollen grain in polar view, distal side. **H.** Pollen grain in polar view, proximal side. **I.** Close-up of apex, proximal side. **J.** Close-up of central polar area, distal side. Scale bars – 10 µm (A–C, F–H), 1 µm (D, E, I, J).

IPUW 7513/228 and IPUW 7513/229 (Figure 6).

**Figure 7.**
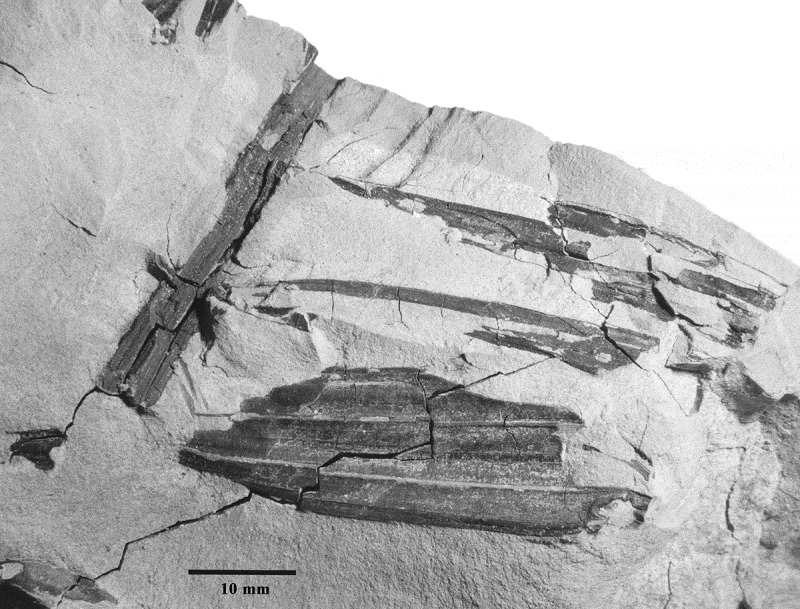
Fossil leaf fragment from locality CH41, specimen 9A (CH41-9A), collected at 74 m above the base of the measured Guang River section (Figure 2).

#### Type locality

Guang River, Chilga, North Gondar Zone, Amhara Region, north-western Ethiopia; *c*. 12° 30′ N, 37° 7′ E.

#### Stratigraphy

Chilga strata (Figure 2).

#### Age

Late Oligocene, 28–27 Ma (Kappelman et al. 2003).

#### Species epithet

After the extant species, *Sclerospermaprofizianum*, that also shows a clear microreticulate to perforate sculpture in SEM.

#### Description

Pollen, monad, heteropolar, P/E ratio oblate, outline straight-triangular in polar view, bean-shaped in equatorial view (convex distal face versus concave proximal face); equatorial diameter 23–35 µm in LM, 21–29 µm in SEM, polar axis 9–12 µm in LM; triporate, pori positioned sub-apically on the distal polar face, pori elliptic, 6.0–8.0 µm in diameter, pori equipped with opercula; exine 1.7–2.5 µm thick in LM; pollen wall semitectate to tectate; sculpture reticulate in LM, microreticulate to perforate in SEM; distal face microreticulate to perforate with broad muri and elliptic to polygonal lumina or circular to slit-like perforations, 50–65 lumina/perforations per 100 µm^2^ at central distal face, free-standing columellae rare, up to four nanogemmae free-standing columellae per lumina when present (SEM); proximal face microreticulate to perforate, lumina/perforations elliptic to circular to polygonal or slit-like, free-standing columellae rare, up to four nanogemmae free-standing columellae per lumina when present, central polar area and mesoporium microreticulate to perforate, sculpture becoming nanoreticulate to perforate towards apices; opercula with nanoverrucate to granulate sublayer and a microreticulate supra-layer (SEM).

#### Remarks

The morphology of this fossil pollen type also clearly places it within *Sclerosperma* (Table I). Of the four extant pollen types it is most similar to *S.profizianum* Type A and *S.profizianum* Type C (see Grímsson et al. 2018), and could be considered an intermediate form between those two extant pollen types. The *S. protoprofizianum *sp. nov. differs from the *S.profizianum* Type A and Type C pollen in both outline and size. The *S.protoprofizianum* sp. nov. pollen has only been recovered showing a straight-triangular outline in polar view, but both *S.profizianum* Type A and Type C pollen is also concave-triangular or convex-triangular. The *S. protoprofiziana* sp. nov. pollen is smaller than both the *S.profizianum* Type A and Type C pollen. The sculpture at the central distal face of *S.protoprofizianum* sp. nov. ranges from microreticulate to perforate and the pollen wall is therefore considered semitectate to tectate versus microreticulate and semitectate in *S.profizianum* Type A versus perforate and tectate in *S.profizianum* Type C. The *S.protoprofizianum* sp. nov. pollen has 50–65 lumina/perforations per 100 µm^2^ at the central distal face, but *S.profizianum* Type C has 45–55 per 100 µm^2^ and *S.profizianum* Type A has only 35–45 per 100 µm^2^. Also, outline of the lumina in *S.protoprofizianum* sp. nov. is varying and overlapping with that observed in both *S.profizianum* Type A and Type C. The supra-layer of the operculum is clearly microreticulate in *S.protoprofizianum* sp. nov., but reticulate in *S.profizianum* Type A and perforate in *S.profizianum *Type C.

As mentioned above, the scant fossil record (see Discussion) makes it impossible to compare these fossil pollen grains to fossil material other than the *Sclerosperma protomannii* sp. nov. described herein. The two new fossil taxa are easily distinguished using either LM or SEM. The *S*. *protomannii* sp. nov. pollen appears coarsely reticulate in LM, but the *S. protoprofiziana* sp. nov. pollen is very finely reticulate and usually smaller. Also, the *S.protoprofizianum* sp. nov. pollen is straight-triangular in outline, but often with the apices infolded towards the proximal side, giving the pollen a hexagonal appearance. In SEM, the *S. protomannii* sp. nov. pollen is mostly reticulate with 10–20 lumina per 100 µm^2^ at central distal face, but the *S.protoprofizianum* sp. Nov. pollen is microreticulate to perforate with 50–65 lumina/perforations per 100 µm^2^ at central distal face.

Subfamily Arecoideae Burnett

(Figure 7)

#### Diagnosis

The unarmed leaflets and reduplicate plication indicates that the fossil belongs to either the subfamily Arecoideae or Ceroxyloideae.

#### Specimen numbers

CH41-9A and CH41-9B (part and counterpart).

#### Locality

Sublocality CH41 of the Guang River flora. Guang River, Chilga, North Gondar Zone, Amhara Region, northwestern Ethiopia; *c*. 12° 30′ N, 37° 7′ E.

#### Stratigraphy

Chilga strata (Figure 2).

#### Age

Late Oligocene, 28–27 Ma (Kappelman et al. 2003).

#### Description

A compound palm leaf fragment preserved as a compression, with one leaflet attached to a rachis, and two additional leaflets on the same side that appear to have been attached to the same rachis. Leaflets and rachis are exposed. Venation indicates the leaflets are reduplicate, with splitting apparently having been along the minor abaxial ribs. There are three, more prominent, adaxial ribs preserved on the largest of the leaflets, but the specimen is incomplete and this is a minimum number. In addition, the dimensions of the original leaf, including its rachis and leaflets are unknown. The fragmentary, largest leaflet on the specimen measures approximately 40 mm long by 16 mm wide.

#### Remarks

The unprotected leaflets and reduplicate plication indicates the fossil belongs to either the subfamily Arecoideae or Ceroxyloideae. Extant Ceroxyloideae palms are absent from the African continent, but the genus *Ravenea* is endemic to Madagascar and the Comoros (Dransfield & Beentje 1995), and the monotypic *Oraniopsis* is endemic to Queeensland, Australia (Dransfield et al. 2008). Both *Ravenea* and *Oraniopsis* differ from the fossil by having a prominent midrib on the leaflets that is thicker and wider than exmedial primary veins. Among living Arecoideae, Africa has very few genera and species: those found on the mainland are *Elaeis, Podococcus*, and *Jubaeopsis*, all of which are monotypic, and *Sclerosperma* with three species as discussed herein. The monotypic genus, *Dypsis*, occurs on the island of Pemba (Dransfield 1988; Govaerts & Dransfield 2005). The fossil resembles *Elaeis, Podococcus*, and *Dypsis* in having pinnately-compound leaves with unarmed leaflets, but differs from *Elaeis* and *Dypsis* leaflets which have prominent midribs, and from *Podococcus* leaflets which are distinctively wedge-shaped (Dransfield et al. 2008). *Jubaeopsis* leaflets possess a prominent single reduplicate fold, also unlike the fossil. If the fossil accurately represents leaflets and rachises occurring on the whole plant, then *Sclerosperma* is the only extant African palm taxon that resembles the fossil leaflet material.

## Discussion

### Fossil record of *Sclerosperma*

The macrofossil and pollen records of palms (Arecaceae) have been summarised in detail by e.g. Muller (1981), Harley (1996, 2004, 2006), Harley and Baker (2001), Pan et al. (2006), Dransfield et al. (2008) and Martínez et al. (2016). These summaries show that there have been several fossil pollen taxa (e.g. *Constantinisporis* Belsky, *Dorreenipites* Biswas, *Retitrilatiporites* Misra, Singh et Ramanujam, *Trilatiporites* Ramanujam, *Victorisporis* Belsky, Boltenhagen & Potonie; Misra et al. 1996), from the Upper Cretaceous to late Cainozoic (Miocene), affiliated with extant *Sclerosperma*. Most of these affiliations were rejected by Harley and Baker (2001) and Harley (2004, 2006). The LM and SEM based pollen morphology of extant *Sclerosperma* presented by Grímsson et al. (2018) also supports the rejection of any affiliation of these fossil pollen grains with extant *Sclerosperma*, and excludes all previous fossil pollen accounts except for a single record. The only convincing fossil pollen record of *Sclerosperma*, prior to this study (Table II), is that from the late Miocene of Senegal (West Africa) by Medus (1975, plate 4, figures 2 and 3). The macrofossil record is also scant (Table II), with only a single find including leaves with cuticles presumed to be of Miocene age from the DRC (Lakhanpal 1966, plate 1, figure 1; plate 2, figure 1; plate 3, figures 1 and 2). This provides little information on which to base palaeophytogeographic interpretations, but the Chilga fossils demonstrate that *Sclerosperma* can be traced back to the late Oligocene of East Africa, its earliest fossil record, and that it most likely had a tropical transcontinental distribution across Africa during the late Oligocene/early Miocene.

### Origin and evolution of *Sclerosperma* pollen

Molecular phylogenetic studies estimate that the Arecaceae evolved (stem node/lineage) and started to diverge (crown node age) around 120 to 100 Ma (e.g. Janssen & Bremer 2004; Couvreur et al. 2011; Baker & Couvreur 2013a, 2013b; Hertweck et al. 2015). This scenario seems to fit quite well with the age of the earliest palm fossils summarised in Harley (2006) and Dransfield et al. (2008), that date back to the Aptian (125–113 Ma; ICS 2017) and/or Albian (113–100 Ma; ICS 2017). According to these authors palm fossils apparently become more frequent and diverse in Coniacian (*c*. 90–86 Ma; ICS 2017) sediments, and especially in strata of Maastrichtian age (*c*. 72–66 Ma; ICS 2017). Molecular dating analyses also suggest the crown node age of subfamily Arecoideae to be 75–70 Ma (Baker & Couvreur 2013b), and that *Sclerosperma* diverged from *Orania*, its potential sister genus in the early Oligocene, 35–30 Ma (see figure 2 in Roncal et al. 2010, and figure 4 in Baker & Couvreur 2013b), at the latest. This allows for a potential intrageneric divergence period in *Sclerosperma* of about 20 million years prior to the deposition of the Chilga fossils. It is interesting that the two genera, *Podococcus* G. Mann et H. Wendl. (tribe Podococceae) and *Orania* Zipp. (tribe Oranieae), believed to be genetically closest to *Sclerosperma*, both produce only monosulcate pollen (Dransfield et al. 2008). A sulcus is the basic aperture type in Arecaceae, and suggests that the last common ancestor of *Orania, Podococcus* and *Sclerosperma* also produced sulcate pollen. Erdtman and Singh (1957) suggested an evolutionary transition in apertures from sulcate to trichotomosulcate, to triporate in *Sclerosperma*, showing drawings of non-mature *Sclerosperma* pollen with an apparent ‘crypto-trichotomosulcus’. No such features were observed by Grímsson et al. (2018) in their study on extant *Sclerosperma* pollen, but this kind of evolutionary trend seems quite plausible and was also recognised by Harley (1999) and Harley and Baker (2001, figure 109). Still, the morphology of the earliest *Sclerosperma* pollen, and documentation of early morphological evolutionary trends in the pollen of this genus, will only be possible through the study of Eocene to Oligocene fossils from (palaeo) tropical Africa. Based on the fossil material presented here, and the LM and SEM pollen morphology of extant *Sclerosperma* by Grímsson et al. (2018), it is clear that most sculpture features observed in the extant taxa were already present during the late Oligocene. The fossil material from the late Oligocene, 28–27 Ma (Kappelman et al. 2003), of Chilga shows that African *Sclerosperma* had already diverged into at least two clearly defined morphological lineages: a coarsely reticulate lineage leading to both *S. mannii* and *S. walkeri*, and a microreticulate/perforate lineage leading to the *S. profizianum* (pollen Type A, B and C) species complex.

### *Sclerosperma* and the palaeovegetation – a match or mismatch

There are several reports of macrofossils, and a few about fossil pollen and spores, recorded from the Chilga sediments exposed along the Guang River. The macrofossil record is derived from two main strata shown in Figure 2: one is the dated ash bed (actually a series of repeated ashfalls) at 96–97 m above the base of the section, and the other is the stratum that produced the unknown Arecoid palm leaf fragment discussed above (at 30.5 m above the base of the measured section; Figure 2). The ash fall (paleo) surfaces were apparently quickly colonised by ferns and allies including *Blechnum* (Blechnaceae), *Marsilea* (Marsileaceae), *Acrostichum* (Pteridaceae), *Salvinia* (Salviniaceae), *Cyclosorus* (Thelypteridaceae) and *Equisetum* (Equisetaceae) (García Massini et al. 2006, 2010; García Massini 2009). Monocots found in some horizons among the ash deposits included *Pandanites* (Pandanaceae), *Typha* (Typhaceae), and palms including *Hyphaene* (García Massini 2009; García Massini et al. 2010), along with rare dicots. The richly fossiliferous horizon at 30.5 m above the base of the section (Figure 2), also referred to as the Guang River Flora, includes a variety of palm (Arecaceae) macrofossils including *Eremospatha chilgaensis* (subfamily Calamoideae; tribe Calameae; subtribe Ancistrophyllinae), *Hyphaene kappelmannii* (subfamily Coryphoideae; tribe Borasseae; subtribe Hyphaeninae), along with a few other monocots, including *Scadoxus* (Amaryllidaceae), and *Dioscorea wilkinii* (Dioscoreaceae; Pan et al. 2006, 2014; Pan 2007). The dicot record is richer, comprising among others *Sorindeia* (Anacardiaceae), *Alstonia* (Apocynaceae), *Tetracera* (Dilleniaceae), *Macaranga* (Euphorbiaceae), *Afzelia afro-arabica* and *Cynometra chaka* (Fabaceae), *Ocotea* (Lauraceae), *Strychnos* (Loganiaceae), *Cola amharaensis* (Malvaceae), *Eugenia* (Myrtaceae), *Vepris* and *Clausena* (Rutaceae), *Cardiospermum* (Sapindaceae), *Pouzolzia* (Urticaceae), and Sapotaceae (Pan 2007, 2010; García Massini 2009; Pan & Jacobs 2009; García Massini et al. 2010; Pan et al. 2010, 2014; Currano et al. 2011). Many of the macrofossils, all of which are compressions, are well preserved and with cuticles. Detailed analyses of some of these taxa show that their potential modern relatives are typical of tropical to subtropical African riparian forest or flooded forest communities of West, Central, and East Africa (e.g. Pan et al. 2014).

The pollen identified so far has mostly been analysed using LM only. The majority of the taxa mentioned by Yemane et al. (1985, 1987), as well as all the taxa listed by García Massini (2009) and García Massini and Jacobs (2011), are not accompanied by micrographs and therefore excluding the possibility of validation or revision. The micrographs provided by Yemane et al. (1987) and Danehy (2010) suggest that some of the pollen are assigned to incorrect families and genera, or show that the features observed in LM do not allow for a particular species/genus/family affiliation. Still, the palynflora contains many thermophilous components that are typical of tropical to subtropical climates (Yemane et al. 1985, 1987; García Massini 2009; Danehy 2010; García Massini & Jacobs 2011).

Stratigraphic and sedimentological studies of the Chilga basin suggest a heterogeneous late Oligocene landscape, determined primarily by lateral variations in the water table, with shallow gradient rivers affecting vegetation units via erosion, flooding and deposition (Jacobs et al. 2005). Both plant macrofossils and fossil pollen/spores, along with sedimentological and insect damage analysis from different sublocalites within the Chilga basin, reflect varying wetland and mesic forest habitats (floodplain, stream margins, ponds, swamps, bogs, as well as various successional stages of a tropical palaeoforest (Jacobs et al. 2005; Currano et al. 2011; García Massini & Jacobs 2011).

All three extant *Sclerosperma* species have a restricted distribution confined to the Guineo-Congolian Region of West and Central Africa. *Sclerosperma mannii* has a disjunct distribution with a population in Liberia, and from southeast Nigeria southward to the DRC, and as far east as the border area of the DRC and Rwanda (van Valkenburg et al. 2008). This species has also been reported from the island of Bioko (Guinea López 1946). It is found in shrub layers of lowland evergreen rainforests, ranging from vegetation occurring just behind the mangroves, swamp forests, periodically flooded forests to valley bottom forests at higher elevations, and persisting in secondary growth up to 1400 m elevation (van Valkenburg et al. 2008). *Sclerosperma profizianum* has a clear disjunct distribution with a population in southwest Ghana and another in the larger tributary of the Congo River, including the extreme southwest of Gabon (van Valkenburg et al. 2008; Bourobou Bourobou et al. 2016). It is found on relatively dry patches in swampy areas, valley bottom forests or forests that are waterlogged and/or along streams. *Sclerosperma walkeri* occurs in the interior of Gabon and along the lower reaches of the Congo River. It grows in lowland evergreen rainforests, ranging from swamp forests, periodically flooded forests to lower slopes on terra firma, and persisting in secondary growth at 300–400 m elevation. This species appears to prefer terra firma forests as opposed to *S. mannii* (van Valkenburg et al. 2008).

It is interesting that the current ecology of *Sclerosperma* seems to fit perfectly with the visualised palaeoenvironment, habitats and vegetation units during the late Oligocene at Chilga, as suggested by previous studies based on various geological and palaeontological proxies (Jacobs et al. 2005; Currano et al. 2011; García Massini & Jacobs 2011; Pan et al. 2014). During the late Oligocene, *Sclerosperma* was most likely an important component of the paleovegetation, growing along streams, in swampy areas and on most available periodically flooded substrates.

## Conclusion and outlook

The discovery of pollen representing two extinct species of *Sclerosperma* in strata of late Oligocene age in north-western Ethiopia, demonstrates a much wider distribution for this genus in the past, and provides a minimum age for its evolutionary diversification, which led to its unusual triporate morphology. Today, *Sclerosperma* is restricted to the Guineo-Congolian forested regions of West and Central Africa, and is known to prefer edaphically wet habitats. Thus, the genus shows ecological conservatism, as the pollen was found in an organic-rich, lignitic, matrix, and plant macrofossils from other deposits in the Guang River section were interpreted as representing moist and/or seasonally inundated forests similar structurally to those of modern West, Central, and eastern Africa. The primary difference between modern communities that may serve as living analogues, and the Chilga forests is the common presence of palms during the late Oligocene, even in terra firma sediments. Further resolution and the evolution (and extinctions) of African Arecaceae, and their ecological role during the Paleogene, must be resolved through additional palaeobotanical research.
